# Peptides Derived From Mismatched Paternal Human Leukocyte Antigen Predicted to Be Presented by HLA-DRB1, -DRB3/4/5, -DQ, and -DP Induce Child-Specific Antibodies in Pregnant Women

**DOI:** 10.3389/fimmu.2021.797360

**Published:** 2021-12-21

**Authors:** Matthias Niemann, Benedict M. Matern, Eric Spierings, Stefan Schaub, Gideon Hönger

**Affiliations:** ^1^ Research and Development, PIRCHE AG, Berlin, Germany; ^2^ Center for Translational Immunology, University Medical Center, Utrecht, Netherlands; ^3^ Clinic for Transplantation Immunology and Nephrology, University Hospital Basel, Basel, Switzerland; ^4^ Transplantation Immunology, Department of Biomedicine, University of Basel, Basel, Switzerland; ^5^ HLA-Diagnostics and Immunogenetics, Department of Laboratory Medicine, University Hospital Basel, Basel, Switzerland

**Keywords:** HLA, T-cell epitope, T-cell help, antibody formation, pregnancy

## Abstract

Predicted Indirectly ReCognizable Human Leukocyte Antigen (HLA) Epitopes (PIRCHE) are known to be a significant risk factor for the development of donor HLA-specific antibodies after organ transplantation. Most previous studies on PIRCHE limited their analyses on the presentation of the HLA-DRB1 locus, although HLA-DRB3/4/5, -DQ, and -DP are also known for presenting allopeptides to CD4+ T cells. In this study, we analyzed the impact of predicted allopeptides presented by these additional loci on the incidence of HLA-specific antibodies after an immunization event. We considered pregnancy as a model system of an HLA immunization and observed child-specific HLA antibody (CSA) development of 231 mothers during pregnancy by samples being taken at delivery. Our data confirm that PIRCHE presented by HLA-DRB1 along with HLA-DRB3/4/5, -DQ, and -DP are significant predictors for the development of CSA. Although there was limited peptidome overlap observed within the mothers’ presenting HLA proteins, combining multiple presenting loci in a single predictor improved the model only marginally. Prediction performance of PIRCHE further improved when normalizing scores by the respective presenters’ binding promiscuity. Immunogenicity analysis of specific allopeptides could not identify significant drivers of an immune response in this small cohort, suggesting confirmatory studies.

## Introduction

In solid organ transplantation, the recipient’s adaptive immune system is continuously faced with allogeneic human leukocyte antigen (HLA) proteins introduced by the donor organ. Various computational models to predict the different pathways of allorecognition were suggested to estimate the risk of the humoral immune response (1, 2). The Predicted Indirectly ReCognizable HLA Epitopes (PIRCHE-II) algorithm is designed to estimate allorecognition *via* the indirect pathway. It estimates the number of donor HLA protein-derived peptides that are predicted to be presented within the binding cleft of the recipient’s HLA Class II molecules on antigen-presenting cells (2). Recognition by appropriately binding T-cell receptors of T follicular helper cells leads to T-cell activation, supports B-cell proliferation, and thus promotes the production of donor-specific HLA antibodies (DSA) (3).

So far, the studies on PIRCHE-II have mainly taken into account the HLA-DRB1 locus for peptide presentation, as no typing data on other loci were available. The number of HLA epitopes presentable within donor DR heterodimers were previously shown to correlate with the development of post-transplant *de novo* DSA and graft survival in kidney, liver, and heart transplantation (4–7), but also with the development of child-specific HLA antibodies (CSA) in pregnancy (8).

The pregnancy model, where paternal HLA alleles potentially trigger the production of CSA (9, 10), has several advantages over the transplant setting with respect to the impact of HLA immunization: i) children are only haplotype-mismatched, ii) similar time and type of exposure to allogeneic HLA, and iii) though biologically immunosuppressed, HLA antibody development is not modulated by additional exogenous immunosuppression (11).

Today’s gene sequencing technologies, e.g., Next-Generation Sequencing (NGS), provide reliable HLA typing results for all transplant-relevant HLA genes, including the so far rarely considered HLA-DRB3/4/5, -DQA1, -DPA1, and -DPB1 loci well beyond protein resolution level (12, 13). Implementing these loci as additional presenting molecules may allow to refine the PIRCHE-II approach and to clarify i) which donor HLA-derived peptides are present on each of the presenter molecules, ii) whether these peptides and their quantity are HLA Class II locus dependent or independent, and iii) the degree of overlap between peptides presented by HLA-DR, -DQ, or -DP heterodimers.

The aim of this study was to explore correlations between numbers of HLA-derived allopeptides presented by different HLA Class II loci and their association with CSA after pregnancy. Based on these analyses, strategies to aggregate PIRCHE-II presented on HLA-DQ and -DP into the so-far DRB1-restricted PIRCHE scores (PSs) were suggested.

## Materials and Methods

### Population and Sample Collection

A cohort of 231 mother–child pairs was examined. The study was approved by the local ethics committee. All women gave live birth at the University Hospital Basel between September 2009 and April 2011. They had either their first full-term pregnancy or previous children only from the same partner as the current live birth. A blood sample was drawn from the mother between days 1 and 4 after delivery for HLA typing and HLA antibody analysis. The cord blood of the child was obtained immediately after delivery for HLA typing. All mothers were healthy women without prior blood transfusions, transplantations, or miscarriages. Pregnancy was therefore assumed to be the only major sensitization event. High-resolution typing for HLA-A, -B, -C, -DPA1, -DPB1, -DQA1, -DQB1, -DRB1, and -DRB3/4/5 was available for all study participants. HLA antibody analysis by single antigen beads (SAB) was only available for mothers.

The median age of the analyzed mothers was 31 years (Q1 = 28, Q3 = 35). Of 231 cases, 143 (62%) had a first full-term pregnancy, while 88/231 mothers (38%) had at least one prior pregnancy from the same partner as the current pregnancy. The ethnicities (according to country of origin) were as follows: 216 Caucasians (93.5%), 11 Hispanics (4.7%), 2 East Asians (0.9%), and 2 Asians/Pacific Islanders (0.9%).

### High-Resolution Human Leukocyte Antigen Typing

White blood cell-derived genomic DNA was used for HLA typing by NGS. The workflow from NGSgo^®^ was applied for HLA amplification and library preparation (www.gendx.com, GenDx, Utrecht, the Netherlands), followed by sequencing on a MiSeq™ instrument from Illumina^®^ (www.illumina.com, Illumina, San Diego, USA) by utilizing MiSeq V2 reagents with the application of paired-end sequencing (2 × 151 bp). The final HLA allele calling was done using the NGSengine^®^ software from GenDx based on the IMGT/HLA 3.33.0 database.

### Identification of PIRCHE Peptides

**Figure 1:**
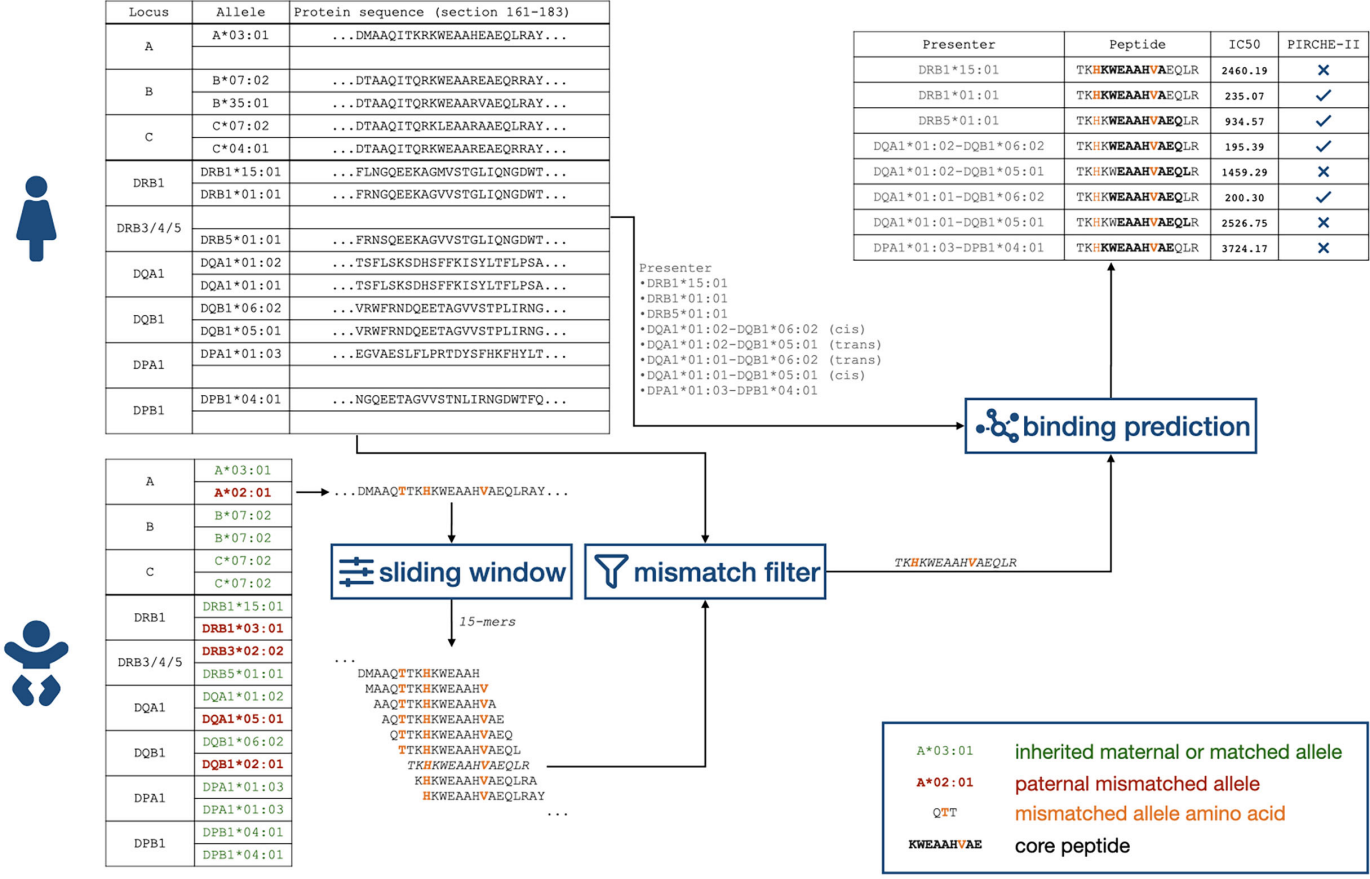
Flowchart of identifying child-HLA-derived peptides presented by HLA Class II proteins. HLA, human leukocyte antigen.

Unambiguous 2-field HLA typing results were extracted from the established NGS results (see above). The PIRCHE algorithm first identified mismatched amino acids (AA) on the linear sequence of allo-HLA proteins. Next, a pool of candidate peptides of 15 AA lengths encompassing the mismatched AA is derived and filtered by the set of maternal self-peptides. The remaining peptides are fed into netMHCIIpan 3.0. This software predicts how these potential ligands align to the binding groove of an HLA Class II molecule, estimates the nonameric binding core, and calculates their HLA-peptide binding affinity (14). For HLA-DR presentation peptide, binding affinities were calculated for HLA-DRB1 and HLA-DRB3/4/5 considering, an HLA-DRA chain invariant in its extracellular domains. For presentation by isotype-matched HLA-DQ and -DP heterodimers, variable alpha chains were combined with beta chains considering cis (i.e., chains present on the same chromosome) and trans (i.e., chains present on opposing chromosomes) allele combinations leading to a maximum of 4 variations of HLA-DQA1-DQB1 and HLA-DPA1-DPB1 per individual (15). Unique core peptides per presenting locus with an affinity of <1,000 nM were considered as PIRCHE (if no different cutoff is stated) (**Figure 1**). The number of such PIRCHE per presenting-locus–presented-locus combination is depicted as PSs.

### Relative PIRCHE Scores Depending on Binding Promiscuity

In the current version of the PIRCHE algorithm, homozygous presenters are potentially underrepresented in the PS, as single-presenting HLA proteins’ PS is only considered once in the overall score calculation. Conversely, peptides presented on two distinct HLA proteins are counted twice by PIRCHE. This behavior has a more notable impact on HLA-DQ and -DP, as homozygous alpha and beta chains only contribute one presenting heterodimer, whereas heterozygous configurations are considered with four presenting heterodimers. HLA Class II proteins were shown to have common binding features as well as allele-specific characteristics, which alter the set of presentable peptides both qualitatively and quantitatively (16–18). To address this, we defined a binding promiscuity score for each HLA Class II protein, which is calculated as the number of all HLA-derived peptides with an IC50 < 1,000 from the IMGT/HLA database (**Supplementary Figure 1**). The mothers’ locus-specific PSs were divided by binding promiscuity score.

### Identification of Child-Specific Human Leukocyte Antigen Antibodies

Post-pregnancy sera were analyzed for child-specific HLA IgG antibodies with LabScreen™ Single HLA Antigen Beads (SAB) (OneLambda Thermo Fisher, Canoga Park, CA, USA) for HLA Class I (iBeads, lot 1) and for HLA Class II (lot 9) according to the manufacturer’s instructions. The CSA^+^ status was assigned if the specific SAB representing the HLA protein of the mismatched paternal HLA allele had a mean fluorescence intensity (MFI) value above the sample-specific biological cutoff defined as the mean of all SAB_self-HLA mother_ + 3 SDs and >100 MFI. In cases where the 2-field HLA typing result revealed an HLA not represented within the SAB panel, the SAB with HLA of the highest AA compatibility (on extracellularly accessible domains, Class I encoded by exons 2/3/4, and Class II encoded by exons 2/3) was considered for CSA analysis.

### Identification of Eplet Match Grade

Antibody-verified (AbVer) Eplets as listed by the Epitope Registry (19) were parsed from the website (epregistry.com.br, version 3.0). Multiple sequence alignments of IMGT/HLA 3.34.0 were considered to identify each allele’s Eplet signature. Based on this data, paternal Eplets, as well as the mothers’ self-Eplets, were identified. Eplet match grade was calculated as the number of Eplets introduced by paternal mismatched HLA, which are not present in a mother’s self-Eplets.

### Statistical Analysis

PS distributions were analyzed with the Shapiro–Wilk normality test. Correlations between locus-dependent PSs were analyzed with Spearman’s rank-correlation coefficient (rho). For correlation analyses of PIRCHE and CSA, individual CSA was pooled by HLA Class with respect to mismatch locus. To adapt for locus-specific PS ranges, PSs were centered in the pooled analyses by subtracting the respective mean and scaled by dividing the centered values by the respective SD. Student’s t-test was applied to compare PIRCHE-II distributions of the overall cohort.

Binding promiscuity of HLA Class II proteins considered HLA-derived peptides from all alleles listed in IMGT/HLA database version 3.44 (20), filled up by the nearest neighbor completion (21), with a netMHCIIpan-predicted IC50 score of below 1,000 nM. In binding promiscuity-corrected analyses, PSs per presenting molecule were divided by the number of presentable peptides per specific presenter.

The second-order Akaike information criterion (AICc) was calculated to compare the quality of prediction of modified PS.

Stepwise logistic regression based on the AICc was applied to find a minimal model of independent variables predicting CSA development. Fisher’s exact test was applied to evaluate the significance of odds ratios of pooled PS.

p-Values of less than 0.05 were considered statistically significant. Bonferroni correction was applied when PS distributions were correlated by presenting-loci and mismatch-loci.

All statistical analyses were performed with R software (R 3.6.1, R Foundation for Statistical Computing, Vienna, Austria). Sequence logos were generated *via* the R “ggseqlogo” library (22). p-Values for differences in peptide position probability matrices were calculated using the R “DiffLogo” package (23).

## Results

### Child Human Leukocyte Antigen-Derived Allopeptide Pool

**Figure 2:**
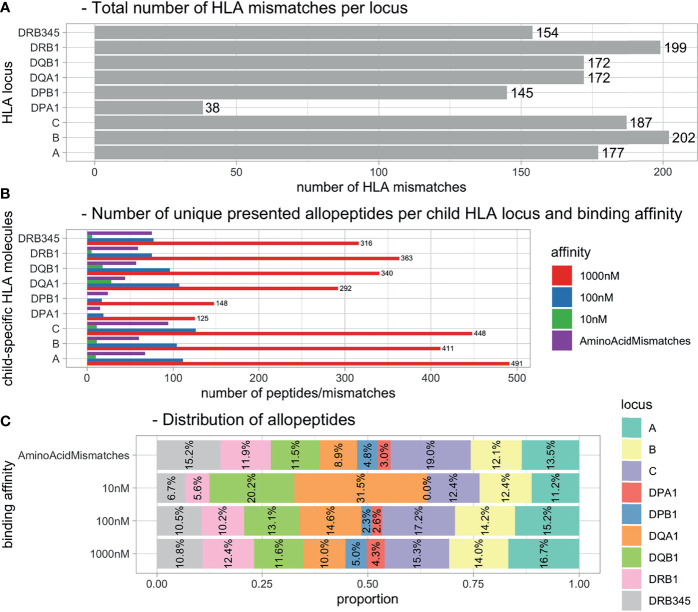
Distributions of HLA mismatches and presented allopeptides. **(A)** Total number of HLA mismatches within all pregnancies. **(B)** Number of unique allopeptides predicted to be presented by maternal HLA considering IC50 threshold. **(C)** Distribution of allopeptide numbers per source locus dependent on IC50 threshold. HLA, human leukocyte antigen.

The 231 mother–child pairs represented 1,446 child HLA mismatches (**Figure 2A**). The PIRCHE algorithm identified a total of 4,174 unique 15-mers, corresponding to 2,618 nonameric cores with a calculated binding affinity of <1,000 nM (**Figures 2B, C**). Limiting on peptides predicted to have a higher binding affinity (decreasing the IC50 threshold by 10-fold to 100 nM and by hundred-fold to 10 nM) revealed a remaining allopeptide pool of 673 peptides (25.7%) for the 100-nM cutoff and 88 peptides (3.4%) for the 10-nM cutoff.

**Figure 3:**
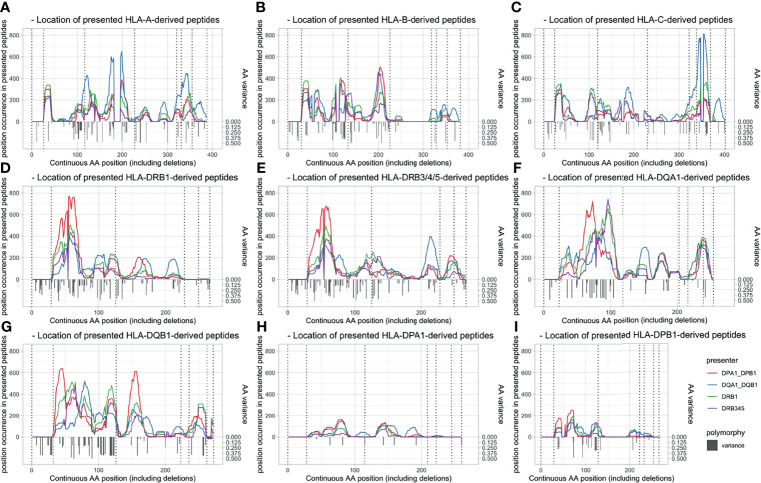
Peptide position of allopeptides dependent on presenting locus (line colors) and presented locus (panel) and polymorphy indication. Amino acid variation is shown with inverted gray bars, and exon limits are indicated by dotted vertical lines. Position (x-axis) is numbered continuously based on the IMGT alignment file, including leader peptides and deletions. **(A)** HLA-A-derived peptides. **(B)** HLA-B-derived peptides. **(C)** HLA-C-derived peptides. **(D)** HLA-DRB1-derived peptides. **(E)** HLA-DRB3/4/5-derived peptides. **(F)** HLA-DQA1-derived peptides. **(G)** HLA-DQB1-derived peptides. **(H)** HLA-DPA1-derived peptides. **(I)** HLA-DPB1-derived peptides. HLA, human leukocyte antigen.

The allopeptides’ positions correspond largely to polymorphic regions on the respective HLA loci, as visualized in **Figure 3**. Presenting loci seem to have an individual preference for the presentation of specific polymorphic regions dependent on the mismatched HLA locus. This is reflected by both the unique presentation capability of single loci (e.g., position 115 in DPA1-derived peptides presented by DQ) and shifts between presented allopeptides’ positions around specific polymorphic regions depending on presenting locus (e.g., position 153 in DQA1-derived peptides). Furthermore, the numbers and frequencies of presented allopeptides were considerably lower for DPA1- and DPB1-derived peptides.

### Presentation of Allopeptides on HLA-DP-, HLA-DQ-, and HLA-DR-Heterodimers

To compare the similarity of PS distributions, PSs aggregated by presented locus were evaluated pairwise. This inter-locus comparison revealed a significant positive correlation between the sum of PIRCHE across HLA-DRB1, -DRB3/4/5, -DQ, and -DP, with Spearman’s rho (r_s_), moderately ranging from 0.38 to 0.51 (p < 0.001, **Supplementary Figure 2**). The correlation with the number of AA mismatches is slightly higher with r_s_ ranging from moderate 0.45 to strong 0.74 (p < 0.001). The sum of AbVer Eplets was moderately correlated with PSs (0.34 < r_s_ < 0.49, p < 0.001) and was strongly correlated with the number of AA mismatches (r_s_ = 0.77, p < 0.001).

**Figure 4:**
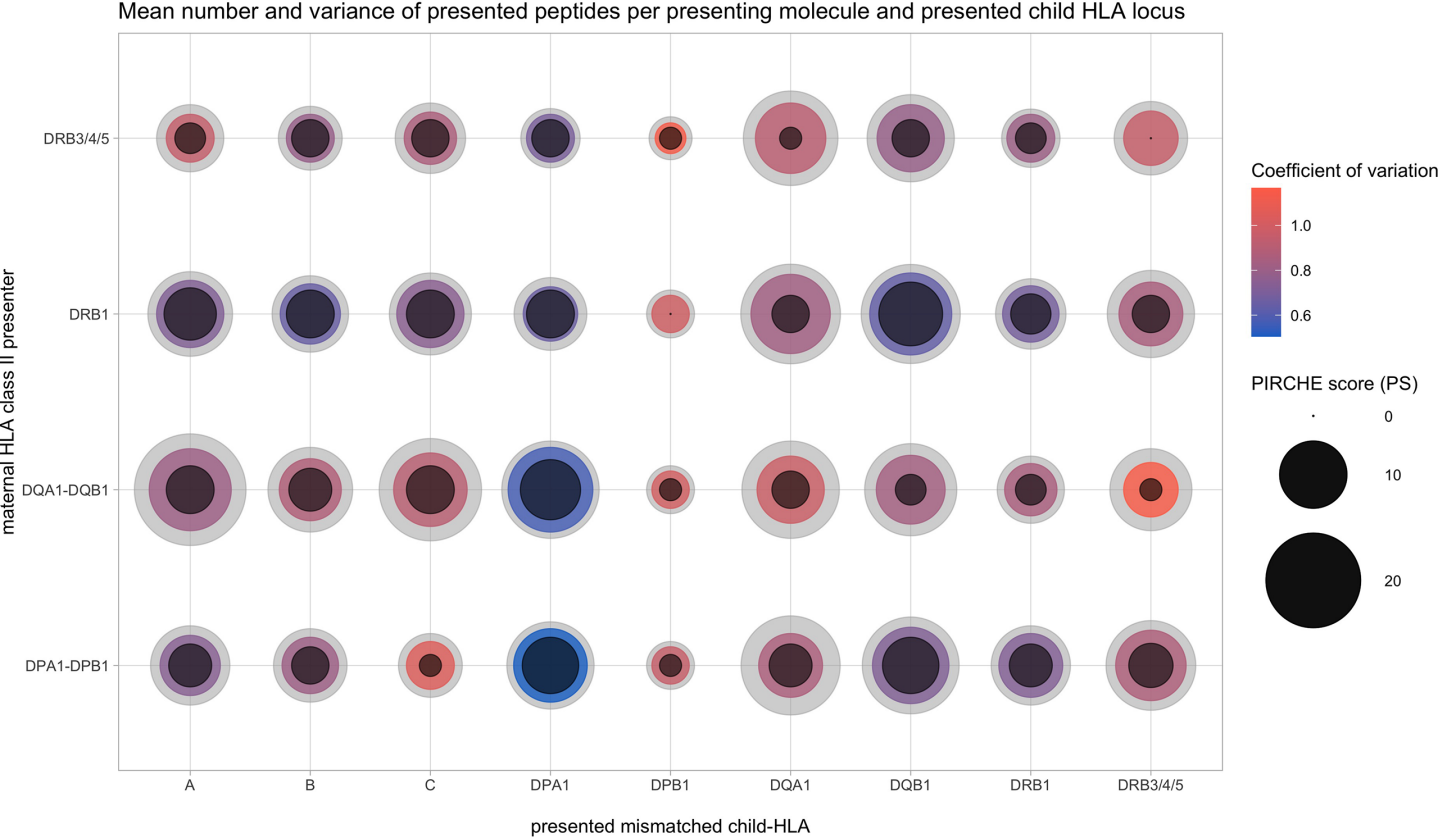
Allopeptide number distributions depending on maternal presenting (y-axis) and paternal presented locus (x-axis). Inner circle size, 25th percentile; colored center circle size, median; outer circle size, 75th percentile; color, coefficient of variation.

Repeating these analyses differentiated by the presented locus also showed significant positive correlations (r_s_ = 0.29 to 0.90, p < 0.001, except for DPA1-derived PIRCHE). This suggests that PSs of certain presenting-locus/presented-locus pairs have considerable overlap in their numerical distributions. This is illustrated by **Figure 4**, indicating median PS, the lower and upper quartiles, and the corresponding coefficient of variation from each presented HLA protein’s locus with respect to the presenting HLA Class II protein. Apparently, HLA-DRB1 and DRB3/4/5 have a numerically similar peptide presentation signature. Within the presented loci, presentation numbers of HLA Class I-derived peptides appear to be more homogeneous. Considering specific combinations of presenting locus and presented locus in **Figure 4**, HLA-DQ and -DP heterodimers are able to present more peptides, possibly related to consideration of both cis- and trans-configurations (e.g., DQ presents HLA-A vs. DRB1 presents HLA-A).

### Cross-Presentation of Certain Allopeptides Within Different Presenter Human Leukocyte Antigen Class II Heterodimers

Next, we focused on the specific allopeptides within each mother–child pair and assessed to which degree they were presented by single or multiple maternal HLA Class II heterodimers. A large fraction of allopeptides (45%–59% depending on locus) were predicted to bind exclusively to one HLA Class II locus, and only a fraction of peptides were predicted to be presented by different loci simultaneously (**Supplementary Figures 3, 4**). However, it has to be acknowledged that identical AA mismatches could be presented by different loci through peptides with an offset of a few AA (**Figure 3H**; e.g., position 192, DPA1-derived peptides, presentation by DP and DQ).

### PIRCHE and Development of Child-Specific Human Leukocyte Antigen Antibodies

Of the 231 mothers, 97 (42%) revealed CSA in their delivery serum (CSA^+^, median = 0, interquartile range (IQR) = 2). Women with previous pregnancies were more frequently CSA^+^ compared with first-time mothers (first pregnancies, 47 CSA^+^ [32%]; prior pregnancies, 50 CSA^+^ [59%]). PS summed across all considered presenting loci were overall higher in CSA^+^ mothers (mean 344 vs. 290, p = 0.011). PS distributions were compared with respect to presenting loci and child-HLA mismatch loci stratified by CSA induction. Considering *all* mother–child pairs (including matched cases), most of these distributions were significantly different (data not shown). To estimate the additional value of analyzing indirect T-cell epitopes of HLA mismatches on predicting CSA occurrence, PS distributions were evaluated in the subgroups of mismatched mother–child pairs. Without the impact of the HLA-matched group on the CSA^−^ control group and potentially due to the reduced sample size, most correlations did not yield statistical significance, although in all but two distributions, elevated medians can be observed in the CSA^+^ group (**Supplementary Figure 5**).

**Figure 5:**
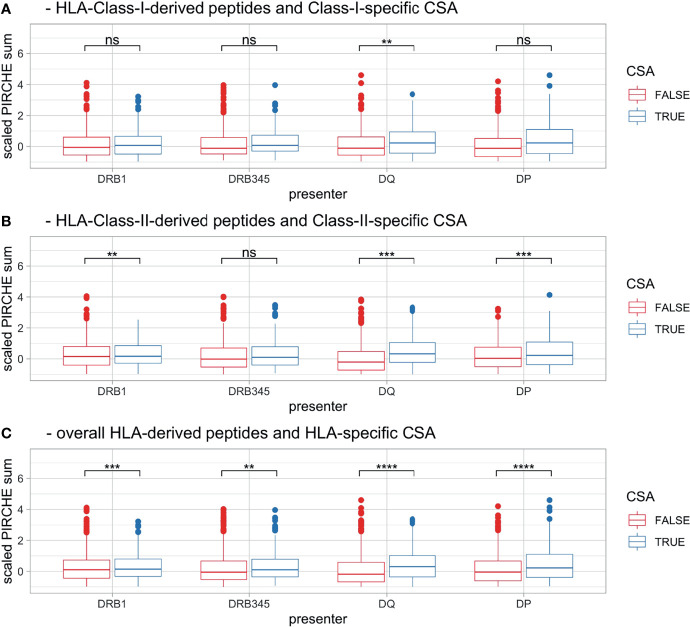
Boxplots [pooling HLA mismatches by **(A)** HLA Class I, **(B)** HLA Class II and **(C)** across all HLA] showing scaled PS, stratified by CSA^+^ incidence (color) and maternal presenting locus (x-axis). Boxplots depict the median (horizontal line), mean (plus), and first to third quartile (box); the highest and lowest values within 1.5× IQR (whiskers) and outliers (circles), respectively. Ns, p > 0.05, **p ≤ 0.01, ***p ≤ 0.001, ****p ≤ 0.0001. HLA, human leukocyte antigen; PS, PIRCHE score; CSA, child-specific HLA antibody; IQR, interquartile range.

In order to increase the statistical power, mother–child pairs were differentiated by mismatched loci and the respective CSA status. These pooled data were aggregated by HLA Class I (HLA-A, -B, and –C; **Figure 5A**), HLA Class II (HLA-DRB1, -DRB3/4/5, -DQA1, -DQB1, -DPA1, and -DPB1; **Figure 5B**), and HLA Classes I and II combined (**Figure 5C**). PSs were scaled as described in the *Materials and Methods* section, taking account of locus-dependent PS distributions. For HLA Class I-derived peptides, only presentation by HLA-DQ was found to be significant (p = 0.006), whereas for HLA Class II-derived peptides, presentation by HLA-DRB1 (p = 0.004), -DQ (p < 0.001), and -DP (p < 0.001) was significantly different between the CSA^+^ and CSA^−^ groups. Furthermore, in the cumulative set of CSA, all Class II presenters had significantly lower PS in CSA^−^ HLA mismatches (DRB1, DQ, and DP, p < 0.001; DRB3/4/5: p = 0.008; **Figure 5C** and **Table 1**).

**Table 1:** Odds ratio of scaled PS of pooled HLA mismatches with respect to CSA^+^ incidence, depending on maternal presenting HLA locus and the corresponding stepwise logistic regression.

	Univariable regression (regular score)			Multiple logistic regression (promiscuity-normalized)		
	Odds ratio	CI	Significance	Odds ratio	CI	Significance
DRB1	1.17	1.03–1.32	*	1.22	1.04–1.42	*
DRB345	1.13	1.01–1.27	*			
DQ	1.28	1.14–1.43	***	1.13	0.98–1.31	.
DP	1.28	1.14–1.43	***			

ns, p > 0.05; PS, PIRCHE scores; HLA, human leukocyte antigen; CSA, child-specific HLA antibody.

^.^p ≤ 0.1, *p ≤ 0.05, ***p ≤ 0.001.

To compare the predictive performance of binding strength, peptide subsets considering only the top 25% best and weakest binders were evaluated (p_strong_, IC50 = (0–189) nM; p_weak_, IC50 = [677–1,000] nM). Correlations with the cumulative pooled CSA data were significant considering the respective subsets p_strong_ and p_weak_ (**Supplementary Figure 6**, except DRB3/4/5 presentation with p_strong_). AICcs were lower or indistinguishable for the p_weak_ PS compared with p_strong_ PS (**Supplementary Table 1**, except DP presenting HLA Class I), suggesting no added value of restricting the peptides’ binding affinity.

Although considering only cross-presented peptides was also significantly correlated with CSA response, in particular with HLA Class II, AICcs did not indicate an improved prediction (**Supplementary Table 1 **and** Supplementary Figure 7**).

Binding promiscuity scores as described in the *Materials and Methods* section were widespread (median = 44,621, IQR = 44,646) across the various HLA Class II proteins found in the present cohort (**Supplementary Figure 1**). Correlating the promiscuity-normalized locus-specific PS in the pooled CSA analysis revealed lower p-values over all but one correlation (HLA Class I-derived peptides presented by HLA-DQ; **Supplementary Figure 8**). Respective AICcs were the lowest compared with all previous models in predicting HLA Class II-specific CSA responses and lower or equal to previous models in predicting HLA Class I-specific CSA responses (**Supplementary Table 1**).

### Immunogenic Allopeptides and Their Frequency

**Figure 6:**
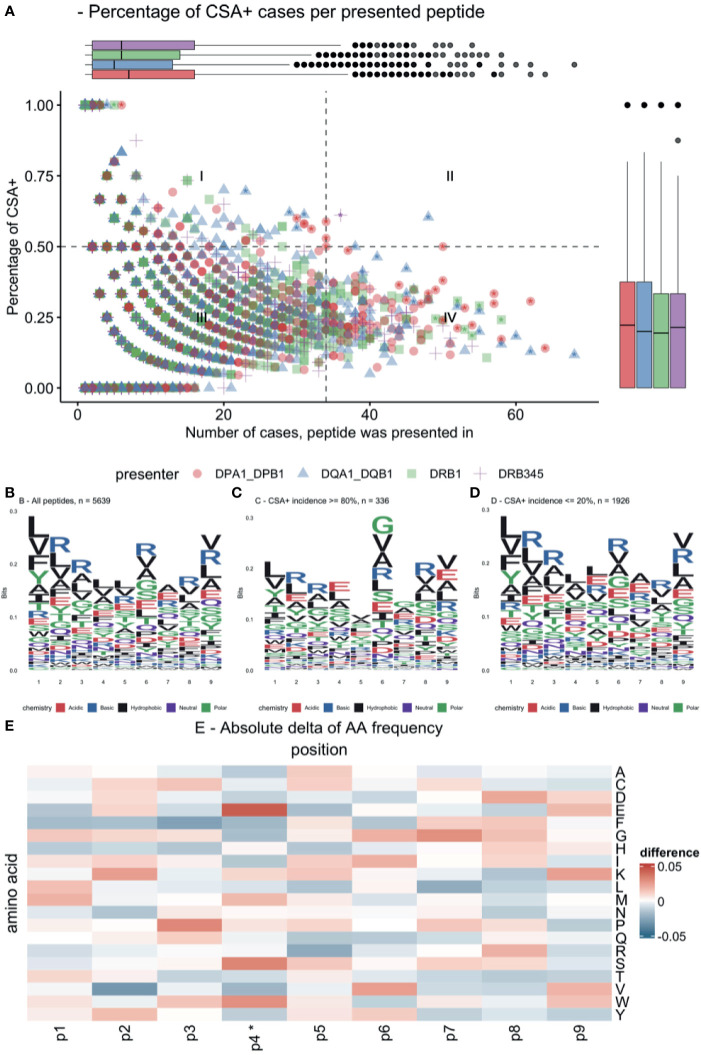
Immunogenic allopeptides and their composition. **(A)** Scatterplot of all presented allopeptides, indicating their relative immunogenicity (y-axis), frequency (x-axis), and maternal presenting locus (color and symbol). Quadrant (I) indicates highly immunogenic, yet rare allopeptides; quadrant (II) indicates frequent immunogenic allopeptides; quadrant (III) indicates rare allopeptides that were not or only rarely co-occurring with CSA; quadrant (IV) indicates frequent allopeptides that were rarely co-occurring with CSA; asterisks indicate peptides, which are characterized in **Supplementary Table 2**. **(B)** Logo plot showing amino acid composition of all core allopeptides, considering identical peptides presented on different loci multiple times. **(C)** Logo plot for allopeptides frequently co-occurring with CSA. **(D)** Logo plot for allopeptides rarely co-occurring with CSA. **(E)** Difference in position probability matrices of peptides in **(C, D)**, *p ≤ 0.05. Amino acid abbreviations in panels **(B–E)** follow IUPAC one-letter codes. CSA, child-specific human leukocyte antigen antibody.

Presentation of specific allopeptides was pooled across cases, and relative CSA^+^ incidence was determined (**Figure 6A**). This revealed that the majority of presented peptides recur in only a few cases (median = 6, IQR = 12). Only 16.8% of presented peptides were associated with a CSA^+^ incidence ≥50%, and 6.0% of presented peptides were always associated with CSA^+^ reaction. Conversely, 30.4% of these peptides were never associated with a CSA^+^ response. Due to individual reaction patterns and multiple testing, these correlations are not statistically significant. However, this plot suggests that certain presented peptides are more immunogenic than others (**Figure 6A**). A subset of 60 peptides with high immunogenicity or high overall frequency was characterized in **Supplementary Table 2**. After correction for multiple testing, no single peptide was found to be statistically significant.

Peptide similarities were evaluated by showing logo plots for peptides with ≥80% CSA^+^ incidence (high-incidence) and peptides with ≤20% CSA^+^ incidence (low-incidence) (**Figures 6B–D**). Subtracting the position probability matrices of high-incidence and low-incidence peptides revealed significant differences in distributions at AA position 4 (**Figure 6E**; e.g., at P4: higher frequency of serine and glutamic acid and lower frequency of phenylalanine and aspartic acid).

### Options to Combine Locus-Specific PIRCHE Scores

Following the aims of the study and based on prior findings, we evaluated the combination of PS of various HLA Class II presenters. Stepwise binomial logistic regression suggested a combination of HLA-DRB1 and HLA-DP as presenting loci of promiscuity-normalized PS for predicting HLA Class I CSA^+^ incidence (data not shown). For HLA Class II CSA^+^, logistic regression suggested HLA-DQ-presented promiscuity-normalized PS as a significant independent variable and dropped HLA-DRB1, -DRB3/4/5, and -DP (data not shown). When HLA Class I and Class II CSA were pooled into a single model, stepwise binomial logistic regression considered promiscuity-normalized PS presented by HLA-DRB1 and -DQ as independent variables contributing to the overall model (**Table 1**).

## Discussion

To the best of our knowledge, this is the first comprehensive investigation on the indirect pathway of allorecognition and its impact on HLA antibody formation based on highly resolved HLA typing data including the HLA-DRB3/4/5, -DQB1, -DQA1, -DPB1, and -DPA1 loci. The study was performed in an immunologically coherent cohort, consisting of non-sensitized women exposed to a similar immunological event. Conversely, transplant recipients are often pre-sensitized, represent varying HLA mismatch constellations with their respective donor, and are immunosuppressed by various medications, which all may confound analyses.

We conducted our study on three levels: determination/comparison of i) the numeric degree of allopeptide presentation (i.e., PS) on each of the investigated HLA Class II loci, their origin, and their respective overlap with each of the investigated HLA Class II loci; ii) the impact of elevated PS on CSA^+^ incidence; and iii) the immunogenicity of the identified presented allopeptides and their expected contribution to CSA induction.

With regard to i), the quantity of the assessed PIRCHE peptides, our analysis revealed different presentation signatures of presenting HLA loci and certain preferences for binding of HLA-derived peptides from specific HLA loci (**Figures 2**–**4**). Allopeptides were mostly predicted to be presented on a single presenter HLA locus. Thus, the overlap between allopeptide repertoires presented by multiple HLA loci simultaneously was limited (**Supplementary Figures 3, 4**). This may be explained by a selective evolutionary pressure to prevent pathogen escape from HLA presentation, which optimized HLA by increasing the variety of presentable pathogens (24, 25). The presenting loci showed a moderate-to-strong positive correlation (**Supplementary Figure 2**). However, it should be considered that the number of presented peptides is *a priori* dependent on the mismatched molecule and their respective number of AA mismatches, limiting the differences in potential allopeptide repertoire sizes.

With regard to ii), our data confirm previous reports about the correlation between the number of HLA-derived peptides being presented on HLA-DRB1 and CSA in the pregnancy setting or DSA in the transplantation setting (4, 8, 26). Moreover, we showed correlations between numbers of HLA-DRB3/4/5-, -DQ-, and -DP-presented HLA-derived peptides and HLA antibody responses (**Figure 5** and **Table 1**). The overall locus-independent correlation with CSA suggests that all loci are likely biologically meaningful in the presentation of indirect T-cell epitopes. We could not confirm the hypothesis that allopeptides are more immunogenic if cross-presented or have a stronger binding affinity to the HLA molecules. Neither cross-presentation nor high binding affinity was significantly more predictive for CSA^+^ response compared with unique presentation and weak binding, respectively (**Supplementary Table 1 **and** Supplementary Figures 6, 7**), the latter supporting with previous reports by Lachmann et al. (4).

Our study showed that normalizing PS based on the respective presenter’s overall binding promiscuity improves the prediction quality (**Supplementary Figures 1, 8 **and** Supplementary Table 1**). The normalization also positively impacted the representation and comparability of homozygous recipients. Consequently, the PIRCHE platform added promiscuity-normalized PS to their output data.

With regard to iii), the immunogenicity of the involved allopeptides, only a few peptides were frequently presented that were also highly associated with CSA response. Partly, this may be explained by the high number of individual constellations of maternal and paternal HLA haplotypes. We could identify peptides that were frequently presented by HLA of CSA^+^ mothers, opening the opportunity to confirm their impact in larger cohorts. However, it has to be acknowledged that presentable allopeptides are so numerous and individual that correcting for multiple testing prevents reaching significance levels.

Our visualizations of the allopeptides’ AA compositions (**Figure 6**), considering the respective peptides’ immunogenicity, lack sufficient normalization by HLA supertypes (27) to clearly identify critical AA positions and differences in their configuration. However, this indicates further potential in the analysis of allopeptides’ AA composition to refine our understanding of T-cell recognition and immunogenicity in the transplantation setting.

Ultimately, stepwise logistic regression suggested a minimal model considering promiscuity-normalized HLA-DRB1 and -DQ presentation in order to predict CSA^+^ responses (**Table 1**). Noteworthy, HLA Class I CSA was best predicted by HLA-DRB1 and -DP presentation, while HLA Class II CSA were equally well predicted by HLA-DQ alone (**Supplementary Table 1**). Combining PS of multiple presenting loci had only a minor impact on prediction performance. Interpretation of indirect T-cell epitope scores may therefore focus on individual mismatches’ PS considering the respective most efficient presenting locus (HLA-DRB1 for Class I and -DQ for Class II).

As shown previously, predicting DSA after organ transplantation with numerically combined Eplet mismatch numbers and PS is superior compared with the predictions based on the independent scores (26). Alongside further improvements of Eplet matching (28, 29), the herein presented modification of the PIRCHE algorithm thus may improve the combined predictions. However, visualizations of co-occurring Eplets and PIRCHE derived from mismatched HLA indicate further the potential of filtering relevant Eplets or PIRCHE (**Supplementary Figure 9**). Eplet-specific PIRCHE signatures suggest peptides both involved and uninvolved in CSA formation. Due to limited cohort size, multiple testing, and structural codependency of Eplets and PIRCHE, this observation is not statistically significant and suggests a thorough investigation of the relationship by exploring potential aggregation functions.

The relatively low number of investigated cases has to be considered as a limitation of the present study, which required pooling the CSA of these cases. Also, as the timeframe between sample collection (at delivery) and the potential immunological sensitization to non-self HLA is relatively narrow, co-consideration of CSA of IgM type could in theory contribute to a more holistic result (30, 31). Compared with the transplant setting, the time of exposure to foreign proteins is rather limited in our cohort. Thus, longer exposure to highly PIRCHE-mismatched HLA may increase immunogenicity.

Considering pregnancy as a model system to study immune responses to allo-HLA is of great value in particular because of the lack of exogenous immunosuppression. However, a variety of immunoregulatory processes are known to be induced by pregnancy as reviewed by Abu-Raya et al. (11), which may limit child-HLA-specific antibody responses. Despite that only women with no prior record of major alloimmunization events were enrolled in the study and given the presence of soluble HLA in seminal plasma (32, 33), prior HLA-specific immunization, e.g., by unprotected sex (34), cannot be fully ruled out in the present cohort due to the unavailability of pre-pregnancy samples. Conversely, Kakaiya et al. (35) reported an HLA antibody prevalence of only 1.6% in healthy female blood donors, suggesting a limited impact of preformed antibodies on the study.

The informative value of the applied HLA antibody diagnostics remains controversial (36–38). Furthermore, natural glycosylation [as reviewed in (39)] may potentially alter the binding of immunoglobulins to HLA (40). One Lambda iBeads were used for HLA Class I antibody detection to mitigate the impact of denatured HLA (41, 42). However, false reactions of the applied assay may systematically confound epitope analyses.

PS for HLA-DQ and -DP consider both cis and trans combinations of alpha and beta chains, following an approach of maximum sensitivity. Although HLA heterodimers are reported to be predominantly present in a cis combination (43), there is also evidence for trans-encoded heterodimers being associated with T-cell activation (44). As it is well known not all trans combinations form stable heterodimers (43), PIRCHE predictions for HLA-DQ and DP may benefit from an inclusion of an HLA heterodimer stability predictor.

In conclusion, the immunologically pristine cohort allowed to refine the PIRCHE model considering high-resolution HLA typing of HLA-A, -B, -C, -DRB1, -DRB3/4/5, -DQA1/-DQB1, and -DPA1/-DPB1. We could show binding promiscuity-normalized PS as presented by HLA-DRB1 or in a combined -DRB1/-DQ model had slightly improved performance in predicting CSA compared with the current PIRCHE-II algorithm. Further research is however warranted to confirm this in the transplant setting and to better understand individual peptides’ immunogenicity.

## Data Availability Statement

The data that support the findings of this study are available from the corresponding author upon reasonable request.

## Ethics Statement

The studies involving human participants were reviewed and approved by Ethikkommission beider Basel (EKBB). The patients/participants provided their written informed consent to participate in this study.

## Author Contributions

MN: conceptualization, data curation, formal analysis, investigation, methodology, software, visualization, writing—original draft preparation, and writing—review and editing. BM: formal analysis and writing—review and editing. ES: writing—review and editing. SS: writing—review and editing. GH: conceptualization, data curation, investigation, resources, supervision, writing—original draft preparation, and writing—review and editing. All authors contributed to the article and approved the submitted version.

## Funding

This study is supported by the Swiss National Science Foundation (grant 32473B_125482/1), Nora van Meeuwen-Hafliger Foundation, research funding from EU Horizon 2020 project code 899708, and research funding from the International HLA and Immunogenetics Workshop Foundation. The authors thank GenDx for financially supporting the study by providing a discount on the typing reagents used. The funder was not involved in the study design, collection, analysis, interpretation of data, the writing of this article or the decision to submit it for publication.

## Conflict of Interest

MN works for PIRCHE AG, which develops and operates the PIRCHE web service. The UMC Utrecht has filed a patent application on the prediction of an alloimmune response against mismatched HLA. ES is listed as an inventor on this patent.

The remaining authors declare that the research was conducted in the absence of any commercial or financial relationships that could be construed as a potential conflict of interest.

## Publisher’s Note

All claims expressed in this article are solely those of the authors and do not necessarily represent those of their affiliated organizations, or those of the publisher, the editors and the reviewers. Any product that may be evaluated in this article, or claim that may be made by its manufacturer, is not guaranteed or endorsed by the publisher.

## Abbreviations

CSA, child-specific HLA antibodies; DSA, donor-specific HLA antibodies; HLA, human leukocyte antigen; MFI, mean fluorescence intensity; PIRCHE-II, Predicted Indirectly ReCognizable HLA epitopes presented by HLA Class II; PS, PIRCHE score; SAB, single antigen beads.
