# Supplementary material for: Peptides Derived From Mismatched Paternal Human Leukocyte Antigen Predicted to Be Presented by HLA-DRB1, -DRB3/4/5, -DQ, and -DP Induce Child-Specific Antibodies in Pregnant Women
Source: Front Immunol. 2021 Dec 21;12:797360. doi: 10.3389/fimmu.2021.797360 (PMC8725048; doi:10.3389/fimmu.2021.797360)
Supplement: Supplementary file 1 [file DataSheet_1.pdf]

Supplementary Material

Supplementary Figures

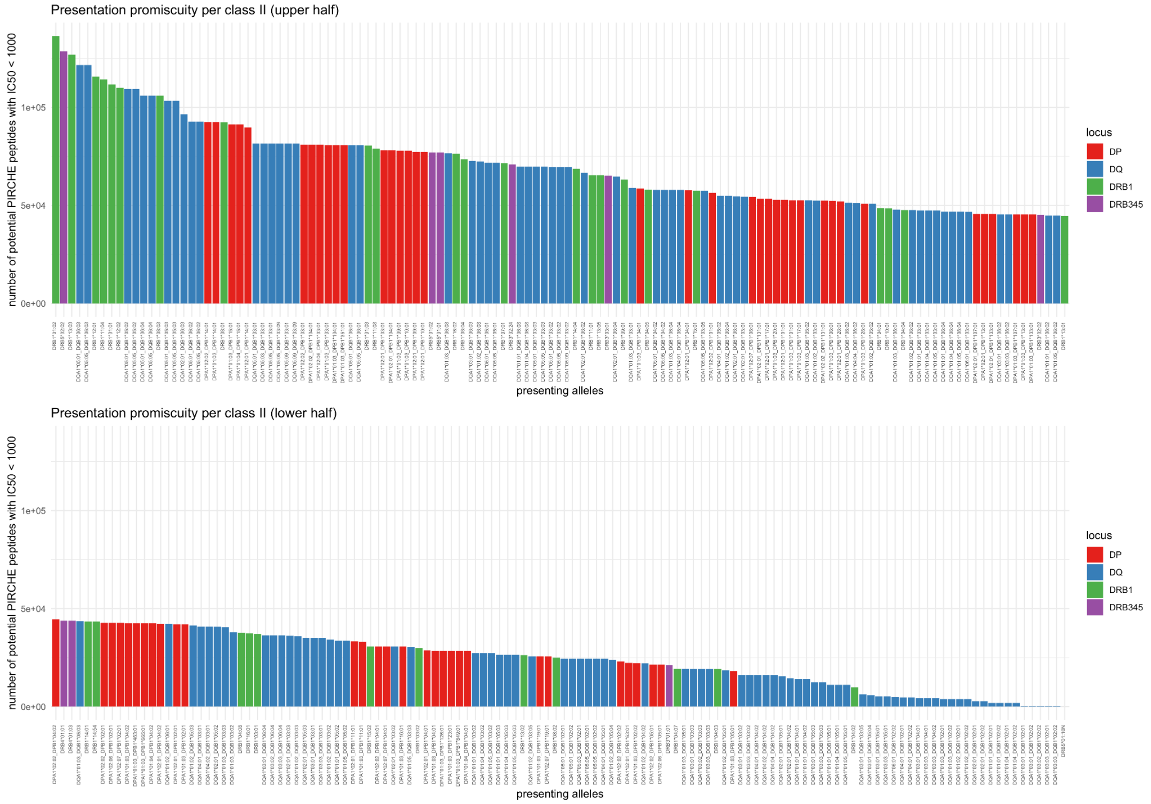

Supplementary Figure 1: Barchart indicating HLA Class II proteins' binding promiscuity. The Y axis depicts the number of HLA-derived peptides predicted having an IC50 score < 1000 nM considering all alleles of IMGT/HLA 3.34. The bars' color corresponds to the presenting molecules HLA locus.

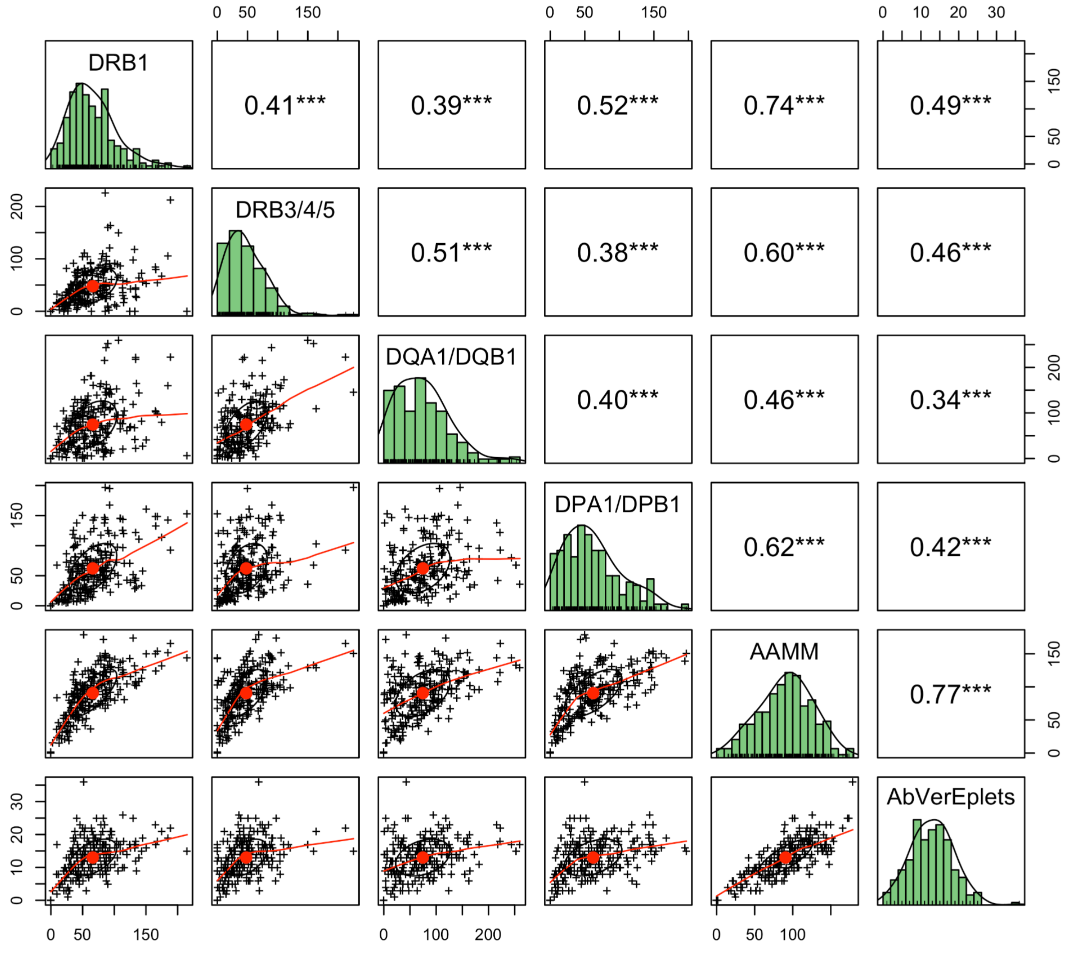

Supplementary Figure 2: Correlation matrix between cumulative PIRCHE being presented by HLA-DRB1, -DRB3/4/5, -DQ and -DP, amino acid mismatch count (AAMM) and antibody-verified Eplet numbers (AbVerEplets). Decimal numbers in the top-right half represent each pair's Spearman's rank-correlation coefficient (rho) with its corresponding significance levels in asterisk notation. ns: $p > 0.05$, *: $p <= 0.05$, **: $p <= 0.01$, ***: $p <= 0.001$

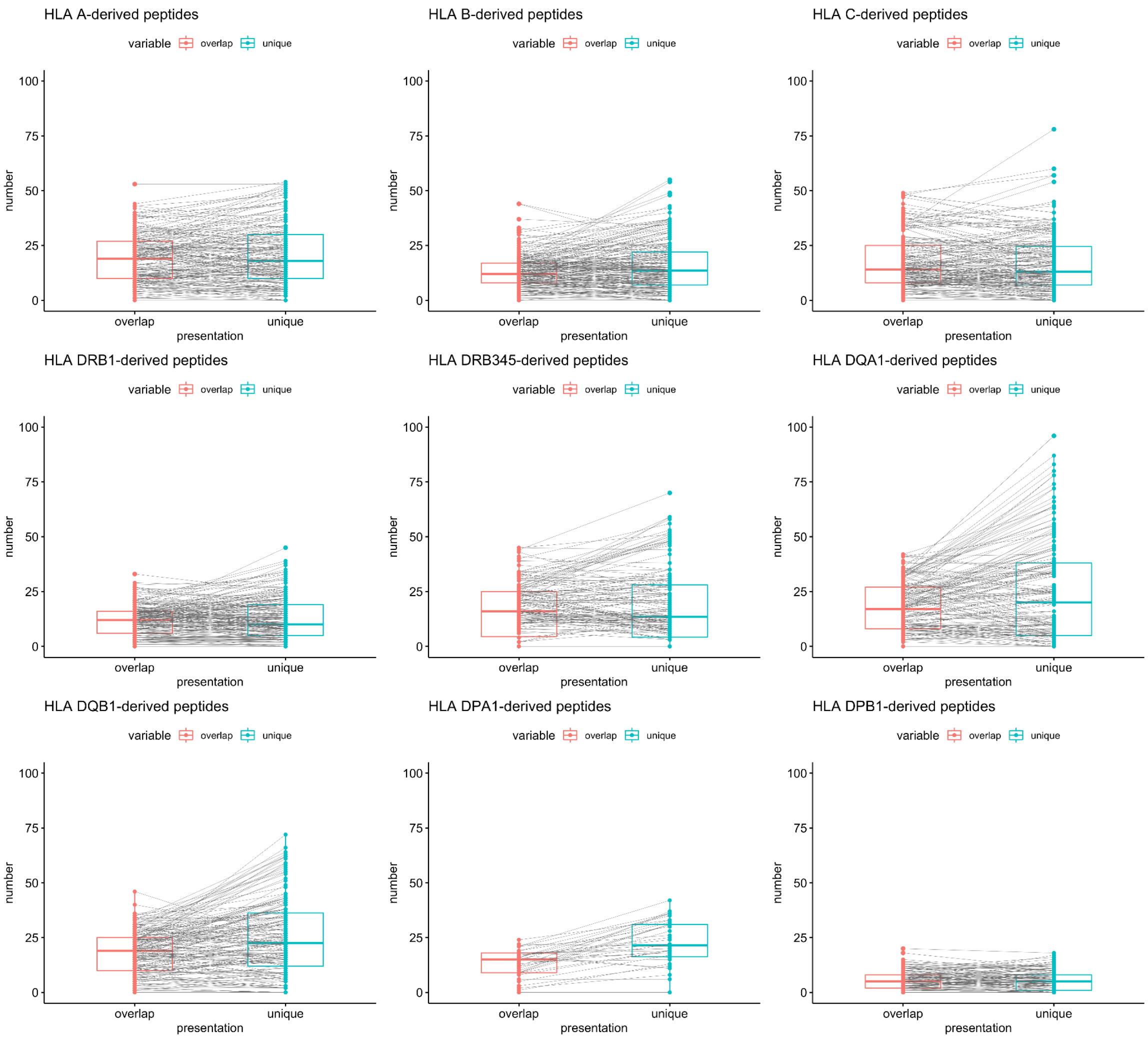

Supplementary Figure 3: Overlapping peptide numbers per locus. Boxes represent the paired number of PIRCHEs per mother either overlapping with multiple presenting loci (red) or being unique per presenter (blue). Connecting lines represent the same case.

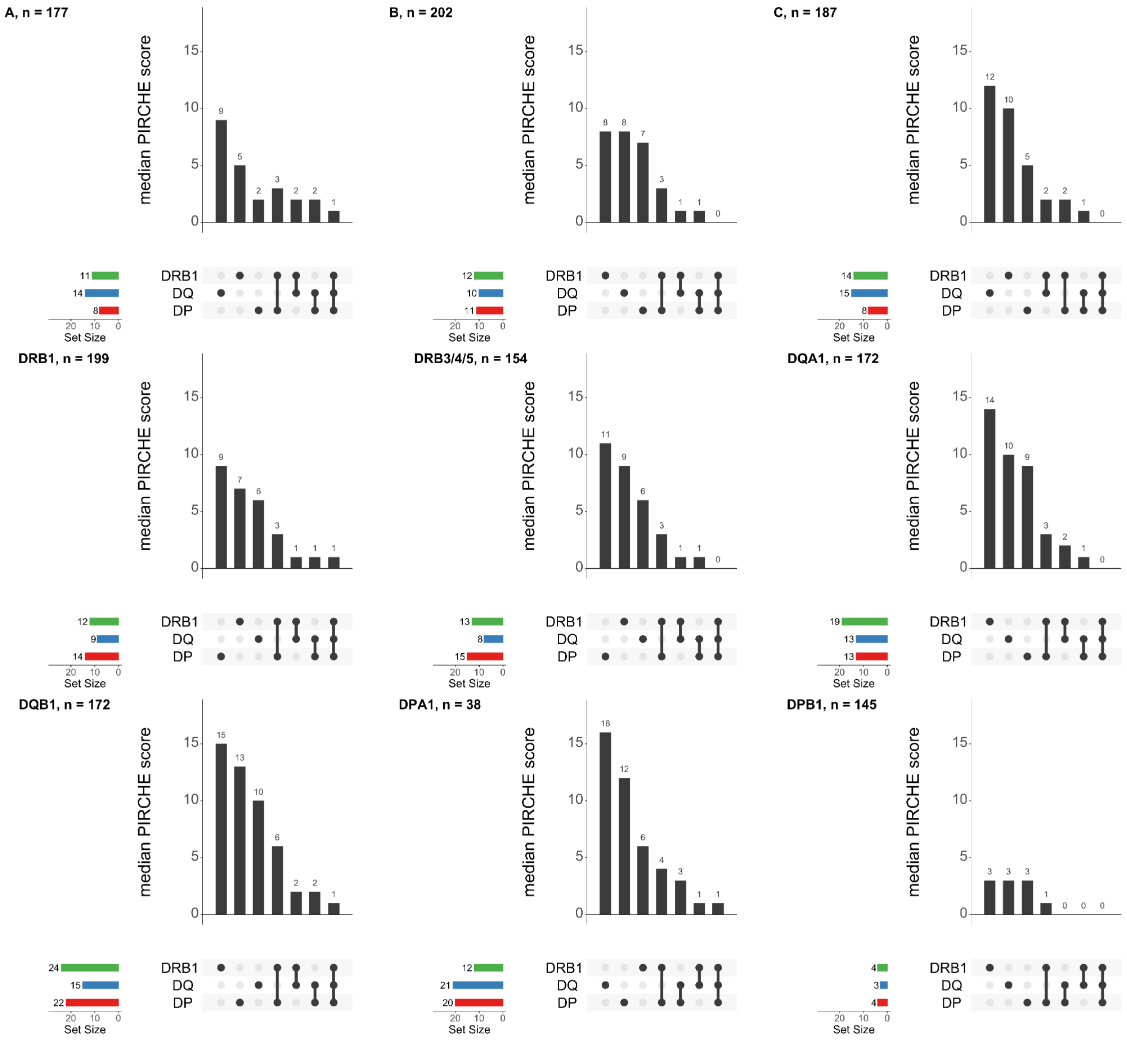

Supplementary Figure 4: Upset plot of median PIRCHE overlap for each presented and presenting loci. DRB3/4/5 was not considered as presenting locus for normalization reasons. Vertical bars represent the median number of PIRCHE per presented locus (panel) considering the set of presenting loci indicated by the dot matrix below the vertical bar chart. Horizontal bars indicate the overall median PIRCHE score per presenting locus as indicated by the dot matrix labels. Plots contain only mismatched cases.

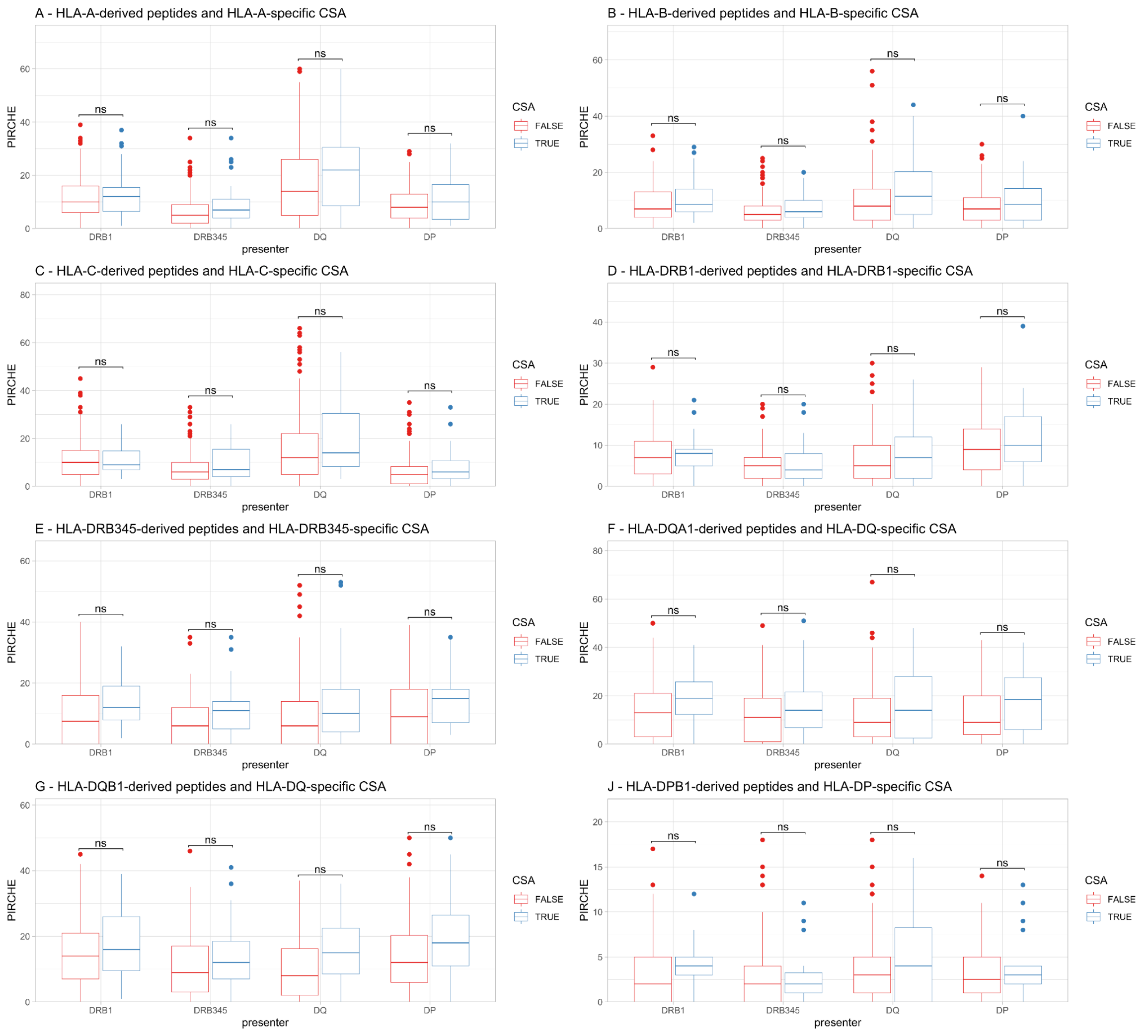

Supplementary Figure 5: PIRCHE score distributions dependent on absence (blue) or presence (red) of DSA depending on presented HLA locus (panel) and presenting molecules' loci (x axis). Box plots exclude matched cases (per panel). Statistical significance determined by Wilcoxon signed-rank test with Bonferroni correction for multiple testing (per panel). ns: p > 0.05, *: p <= 0.05, **: p <= 0.01, ***: p <= 0.001, ****: p <= 0.0001

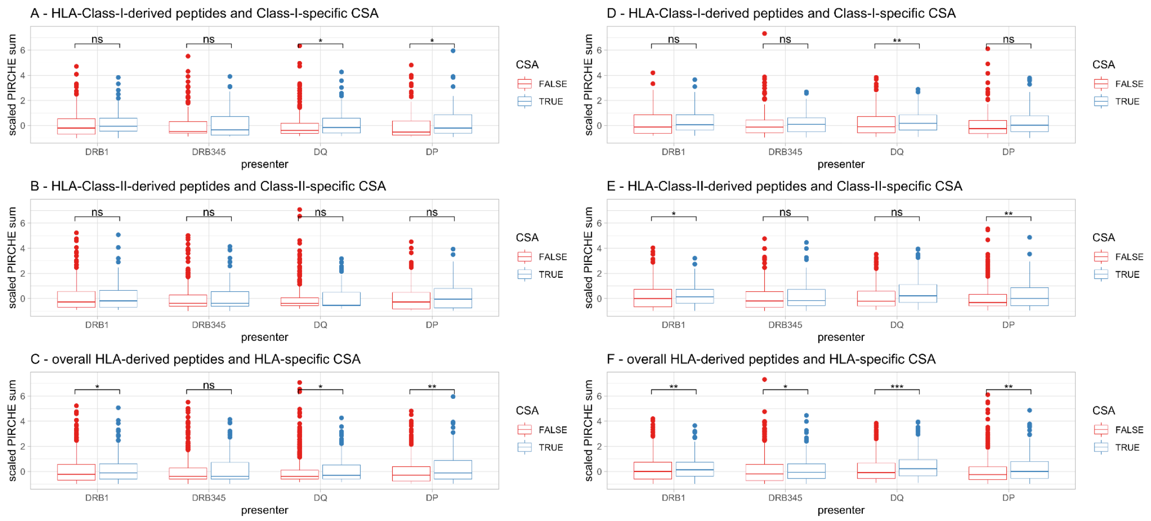

Supplementary Figure 6: Pooled boxplots of scaled PIRCHE score distributions considering HLA Class I (panels A and D), Class II (panels B and E) and overall (panels C and F). Panels A, B and C consider only strong binding peptides with IC50 < 189 nM (i.e. upper quartile), whereas panels D, E and F only consider weak binding peptides with IC50 > 677 (i.e. lower quartile). PIRCHE scores were centered and normalized per presented and presenting locus prior to aggregation into boxplots. Only mismatched alleles were considered, with CSA^{+} mismatches depicted in red and CSA^{-} cases depicted in blue. Box plots exclude matched cases (per panel). Statistical significance determined by Wilcoxon signed-rank test with Bonferroni correction for multiple testing (per panel). ns: p > 0.05, *: p <= 0.05, **: p <= 0.01, ***: p <= 0.001, ****: p <= 0.0001

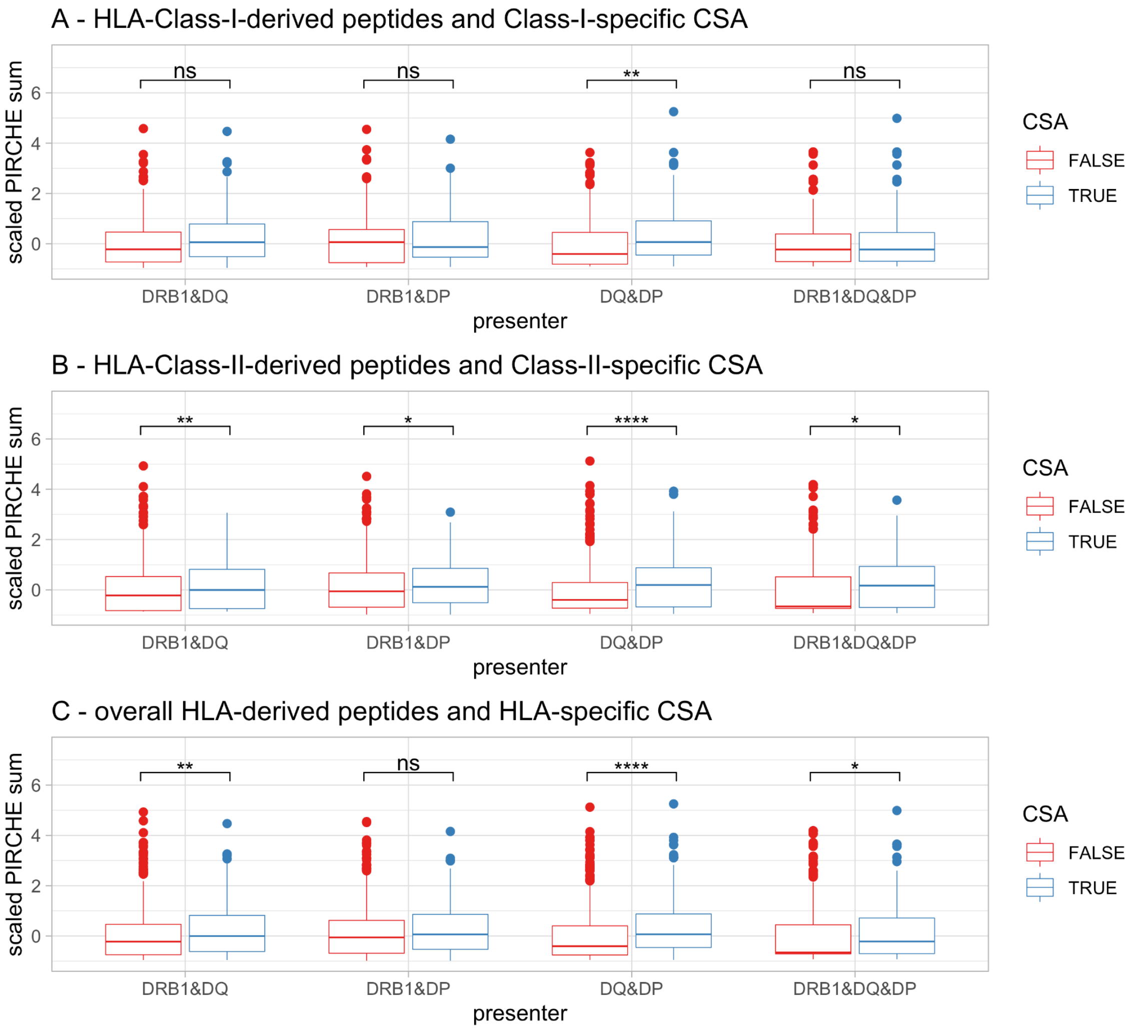

Supplementary Figure 7: Pooled boxplots of scaled overlapping PIRCHE score distributions (i.e. PIRCHE peptides being presented by multiple loci simultaneously) considering HLA Class I (panel A), Class II (panel B) and overall (panel C). The X axis depicts cross-presenting loci. PIRCHE scores were centered and normalized per presented and presenting locus prior to aggregation into boxplots. Only mismatched alleles were considered, with CSA^{+} mismatches depicted in red and CSA^{-} cases depicted in blue. Box plots exclude matched cases (per panel). HLA-DRB3/4/5 was excluded as presenting locus due to lacking normalization. Statistical significance determined by Wilcoxon signed-rank test with Bonferroni correction for multiple testing (per panel). ns: $p > 0.05$, *: $p <= 0.05$, **: $p <= 0.01$, ***: $p <= 0.001$, ****: $p <= 0.0001$

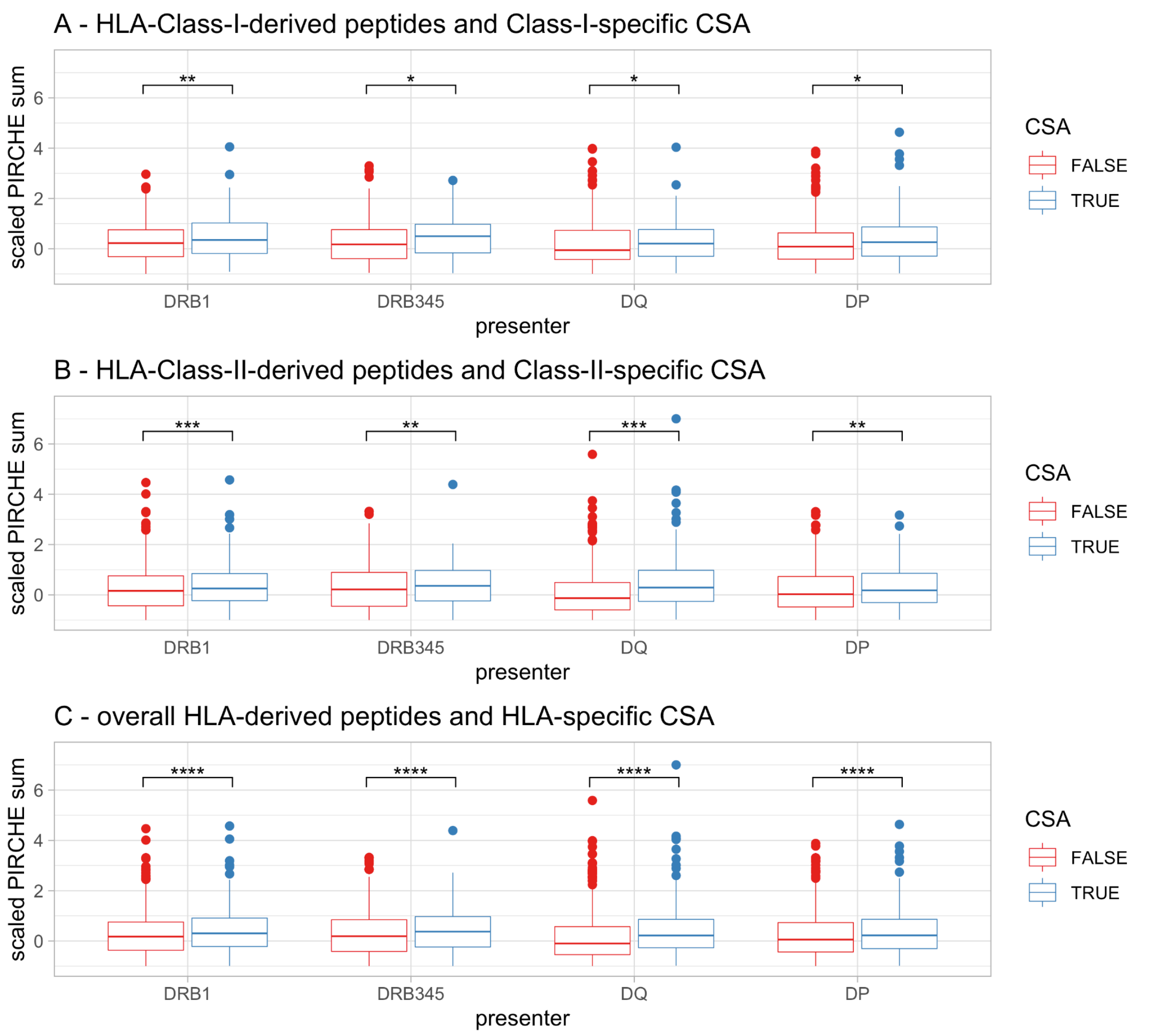

Supplementary Figure 8: Pooled boxplots of scaled, promiscuity-corrected PIRCHE score distributions considering HLA Class I (panel A), Class II (panel B) and overall (panel C). PIRCHE scores were divided by the respective presenters binding promiscuity, centered and normalized per presented and presenting locus prior to aggregation into boxplots. Only mismatched alleles were considered, with CSA^{+} mismatches depicted in red and CSA^{-} cases depicted in blue. Box plots exclude matched cases (per panel). Statistical significance determined by Wilcoxon signed-rank test with Bonferroni correction for multiple testing (per panel). ns: $p > 0.05$, *: $p <= 0.05$, **: $p <= 0.01$, ***: $p <= 0.001$, ****: $p <= 0.0001$

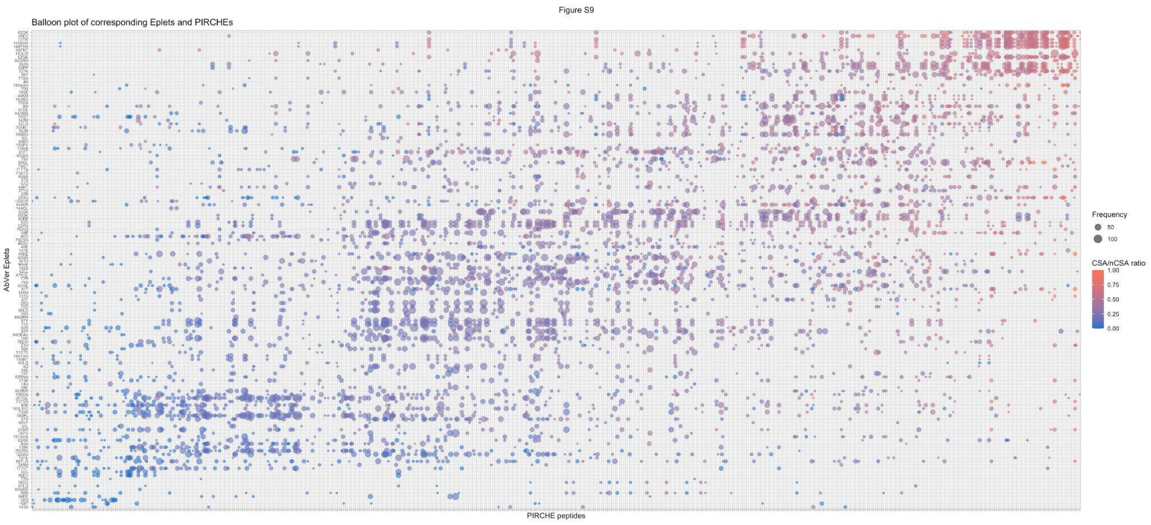

Supplementary Figure 9: Balloon plot showing each mismatch's Eplet (y axis) and PIRCHE (x axis, labels omitted for readability) configuration. Simultaneous cross-presentation by HLA Class II loci counted individually, thus increasing the frequency (size) beyond the number of mismatched cases. The relative immunogenicity (number of CSA^{+} mismatches divided by frequency) represents each circle's color. Only pairs with a frequency > 5 are shown to exclude rare cases. Non-immunogenic peptides (blue) in otherwise immunogenic Eplets (ordered to the top) and immunogenic peptides (red) in otherwise non-immunogenic Eplets (ordered to the bottom) indicate the potential for more sophisticated aggregation of Eplet and PIRCHE matching beyond numeric chaining.

Supplementary Tables

	CL1 CSA				CL2 CSA			
model AICc	DRB1 -> CL1 CSA	DRB345 -> CL1 CSA	DQ -> CL1 CSA	DP -> CL1 CSA	DRB1 -> CL2 CSA	DRB345 -> CL2 CSA	DQ -> CL2 CSA	DP -> CL2 CSA
regular PIRCHE	588.7	587.8	583.7	583.1	888.9	892.0	880.3	881.1
PIRCHE low IC50	590.6	587.7	584.5	585.0	893.0	893.0	894.6	891.7
PIRCHE high IC50	589.1	590.9	585.0	587.8	891.5	892.0	886.8	885.6
promiscuity-corrected PIRCHE	579.7	584.6	587.0	581.3	881.7	885.8	878.0	886.6

	DRB1&DQ -> CL1 CSA	DRB1&DP -> CL1 CSA	DQ&DP -> CL1	DRB1&DQ&DP -> CL1 CSA	DRB1&DQ -> CL2 CSA	DRB1&DP -> CL2 CSA	DQ&DP -> CL2 CSA	DRB1&DQ&DP -> CL2 CSA
cross-presented PIRCHE	586.5	591.2	578.1	589.4	889.1	891.1	881.1	891.6

Supplementary Table 1: Various models' AICc for HLA Class I and HLA Class II respectively.

Core Peptide	Maternal Presenting Locus	Child Locus	ratio	frequency
AYLEGLCVE	DPA1_DPB1	C	1,00	6
AVVATVMCR	DRB1	B	1,00	5
VLAVLAVLA	DQA1_DQB1	C	1,00	4
AADMAAQTT	DQA1_DQB1	A	0,70	23
AGSHTVQRM	DQA1_DQB1	A	0,62	29
QLRAYLEGL	DQA1_DQB1	B	0,61	31
WRFLRGYHQ	DRB345	A	0,61	36
LEGLCVEWL	DQA1_DQB1	B	0,60	48
WRFLRGYHQ	DPA1_DPB1	A	0,60	30
YQFKAMCYF	DPA1_DPB1	DQB1	0,59	34
QLRAYLEGL	DPA1_DPB1	B	0,58	31
LEGLCVEWL	DPA1_DPB1	B	0,58	31
EAAHVAEQL	DQA1_DQB1	A	0,57	30
DFVYQFKAM	DPA1_DPB1	DQB1	0,56	32
FVYQFKAMC	DPA1_DPB1	DQB1	0,53	34

CVEWLRRHL	DPA1_DPB1	B	0,50	50
AGMVSTGLI	DQA1_DQB1	DRB1	0,50	38
EGLCVEWLR	DPA1_DPB1	B	0,50	34
AYLEGLCVE	DPA1_DPB1	B	0,49	37
AYLEGLCVE	DQA1_DQB1	B	0,46	41
LSFYPAEIT	DQA1_DQB1	A	0,42	45
YRENLRIAL	DRB345	B	0,41	39
WEAAHVAEQ	DQA1_DQB1	A	0,41	39
GSIEVRWFL	DPA1_DPB1	DRB1	0,38	50
FLRGYHQYA	DPA1_DPB1	A	0,36	44
AGSHTIQIM	DQA1_DQB1	A	0,35	46
VTRYIYNRE	DPA1_DPB1	DQB1	0,35	46
MAVAKHNLN	DRB1	DQA1	0,35	46
FLDRYFYNQ	DPA1_DPB1	DRB1	0,35	49
RVRFLERHF	DPA1_DPB1	DRB3	0,33	57
EVRWFLNGQ	DPA1_DPB1	DRB1	0,33	48
YELDEAVTL	DQA1_DQB1	DPB1	0,33	45
IFKTNTQTY	DRB1	B	0,31	48
AGLVLLGAV	DQA1_DQB1	A	0,31	48
CGVNLYQFY	DPA1_DPB1	DQA1	0,31	48
FLERHFHNQ	DPA1_DPB1	DRB3	0,31	45
YRESLRNLR	DRB1	B	0,31	52
WRWPEFSKF	DPA1_DPB1	DQA1	0,31	62

LLGLPAAEY	DQA1_DQB1	DQB1	0,30	46
FLGLGLIIR	DPA1_DPB1	DQB1	0,30	57
FKISYLTLL	DPA1_DPB1	DQA1	0,30	47
IKRYNSTAA	DRB1	DQA1	0,29	55
GTVFIIQGL	DQA1_DQB1	DQA1	0,29	52
VVGTVFIIQ	DPA1_DPB1	DQA1	0,28	53
VGEFRAVTL	DQA1_DQB1	DQB1	0,27	55
VFIIQGLRS	DRB1	DQA1	0,24	49
LRKLRGYYN	DRB1	C	0,24	58
YRGILQRRV	DRB1	DQB1	0,24	50
YRGILQRRV	DPA1_DPB1	DQB1	0,24	50
LFIYFKNQK	DPA1_DPB1	DRB5	0,22	54
LIRYIYNQE	DPA1_DPB1	DRB4	0,21	52
FIYFKNQKG	DRB1	DRB5	0,21	53
LAVLGAVMA	DQA1_DQB1	C	0,20	55
FRGILQRRV	DRB1	DQB1	0,19	54
IMGIVAGLA	DQA1_DQB1	C	0,18	60
FRGILQRRV	DPA1_DPB1	DQB1	0,17	58
VLGAVVTAM	DQA1_DQB1	C	0,16	58
YNREEFARF	DPA1_DPB1	DPB1	0,14	64
GLAVLVVLA	DQA1_DQB1	C	0,13	62
LGAVVTAMM	DQA1_DQB1	C	0,12	68

Supplementary Table 2: Subset of 60 core peptides with high immunogenicity or high overall frequency characterized by presenting locus, source locus, immunogenicity and frequency.
